# ENDOSCOPIC SLEEVE GASTROPLASTY - MINIMALLY INVASIVE THERAPY FOR PRIMARY OBESITY TREATMENT

**DOI:** 10.1590/0102-6720201600S10023

**Published:** 2016

**Authors:** Manoel dos Passos GALVÃO-NETO, Eduardo GRECCO, Thiago Ferreira de SOUZA, Luiz Gustavo de QUADROS, Lyz Bezerra SILVA, Josemberg Marins CAMPOS

**Affiliations:** 1 Mário Covas State Hospital, Digestive Endoscopy Department, ABC School of Medicine, Santo André, SP; 2 Hospital das Clínicas, Federal University of Pernambuco, Recife, PE, Brazil.

**Keywords:** Obesity, Bariatric endoscopy, Endoscopic sleeve gastroplasty, Bariatric surgery

## Abstract

**Background:**

Less invasive and complex procedures have been developed to treat obesity. The successful use of Endoscopic Sleeve Gastroplasty using OverStitch^(r)^ (Apollo Endosurgery, Austin, Texas, USA) has been reported in the literature.

**Aim:**

Present technical details of the procedure and its surgical/ endoscopic preliminary outcome.

**Method:**

The device was used to perform plications along the greater curvature of the stomach, creating a tubulization similar to a sleeve gastrectomy.

**Result:**

A male patient with a BMI of 35.17 kg/m^2^ underwent the procedure, with successful achievement of four plications, and preservation of gastric fundus. The procedure was successfully performed in 50 minutes, time without bleeding or other complications. The patient presented mild abdominal pain and good acceptance of liquid diet.

**Conclusions:**

The endoscopic gastroplasty procedure was safe, with acceptable technical viability, short in duration and without early complications.

## RESUMO

**Racional:**

Procedimentos menos invasivos e complexos têm sido desenvolvidos para o tratamento da obesidade. A gastroplastia vertical endoscópica com uso de OverStitch^(r)^ (Apollo Endosurgery, Austin, TX, EUA) já foi relatada com sucesso na literatura.

**Objetivo:**

Apresentar detalhes técnicos do procedimento e seu resultado cirúrgico/endoscópico preliminar.

**Método:**

O equipamento foi utilizado para realizar plicaturas ao longo da grande curvatura gástrica, objetivando tubulização do estômago semelhante a gastrectomia vertical.

**Resultado:**

O método foi aplicado em paciente com IMC 35,17 kg/m^2^, sendo realizadas quatro plicaturas, preservando o fundo gástrico. O procedimento foi realizado com sucesso em 50 min, sem sangramento ou outras complicações. O paciente evoluiu com dor abdominal leve, e teve boa aceitação de dieta líquida.

**Conclusão:**

A realização da gastroplastia endoscópica foi segura, com viabilidade técnica aceitável e reprodutível, com curto tempo de procedimento, sem complicações precoces.

## INTRODUCTION

Bariatric surgery is a well-established procedure in cases of body mass index (BMI) >40 kg/m^2^ or in cases of BMI>35 kg/m^2^ with comorbidities^3^. Currently, gastric bypass and sleeve gastrectomy are two of the most used techniques. Endoscopic methods have become increasingly important in the fight against obesity. However, in cases of grade I and II obesity without comorbidities, the best treatment technique is still uncertain. 

Despite the positive impact of bariatric surgery, only 1% of possible candidates undergo the procedure due to its high cost, limited access, patient preference and related risks^11^. Due to this low rate of performed surgeries and the reduced efficiency of behavioral methods and clinical treatments, less invasive, complex, and lower cost procedures have been developed in order to reach a larger number of patients^5^ ^,^ ^9^ ^,^ ^13^ ^,^ ^14^. 

 Endoscopic sleeve gastroplasty (ESG) using the endoscopic suture system OverStitch^(r)^ (Apollo Endosurgery, Austin, TX, USA) aims to reduce gastric lumen by means of its tubulization, mimicking sleeve gastrectomy surgery and gastric plication^8^ ^,^ ^12^, having recently been approved for use in Brazil. 

The aim of this study was to detail the first ESG procedure performed in Brazil, evaluating technical feasibility, reproducibility and short-term results as primary endoscopic treatment for non-morbid obesity. 

## METHODS

The study protocol for use of suturing devices (Overstitch^(r)^; Apollo Endosurgery, Austin, TX, USA) was approved by the institution's Ethics Committee (Hospital Associação Portuguesa de Beneficência, SP, Brazil, CAAE 1.603.661). The pilot procedure was performed at the Mário Covas State Hospital, ABC School of Medicine, São Paulo, Brazil, following signature of a consent form. 

### Technique

The procedure was performed in an operating room under general anesthesia with orotracheal intubation. The patient was placed in left lateral decubitus, with administration of prophylactic antibiotics (ciprofloxacin). A diagnostic endoscopy was performed before the procedure in order to exclude any possible injuries using a double-channel endoscope, Olympus CV 160 (Olympus Medical Systems Corp., Tokyo, Japan), and insufflation with CO_2_. A specifically developed esophageal overtube (Apollo Endosurgery, Austin, TX, USA) was used to facilitate the repeated and trauma-free passage of the suturing device and to minimize insufflation gas loss, as it features a balloon at its proximal extremity. 

**FIGURE 1:**
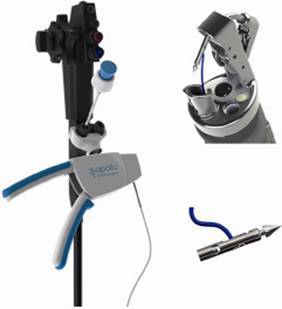
Overstitch(r)Endoscopic Suturing Device; Apollo Endosurgery, Austin, TX, USA

**FIGURE 2:**
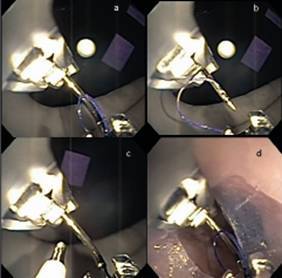
A) Needle and thread assembly; B) needle retraction (preparation for suture stitch); C) tissue Helix(r) device; D) overtube run-through

**FIGURE 3:**
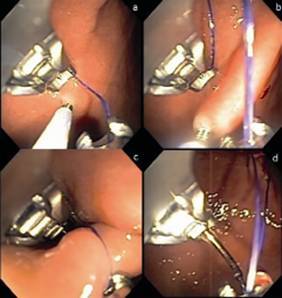
A) Grasping the tissue with the Helix(r) device; B) tissue traction into the device; C) stitch run-through; D) preparation for new suture

OverStich^(r)^ is an endoscopic suturing system which is attached to a double-channel endoscope to perform suturing using a curved needle and polypropylene 2-0 thread to a depth of 15 mm (Figure 1). The drive system is attached to the endoscope handle, while the needle is mounted on the distal end of the device. The needle and the suture are grasped by a system that mimics a needle holder and from where it is possible to deploy, place and remove the thread from the suturing system. The stitches can be either continuous or separate after grasping the tissue with the Helix^(r)^ device (Apollo Endosurgery, Austin, TX, USA), whose function is to drive the tissue into the system and thus allow for full-thickness suturing (Figure 2). At the end of each suture, a knot-closing and cutting system passes through the device's working channel to complete it (Figure 3).

**FIGURE 4:**
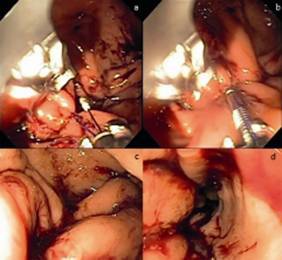
A) Knot pusher; B) tissue retraction and release at end of plication; C and D) Final aspect

The first stitch was placed just above the incisura angularis in U-shaped knots in the following order: anterior wall►greater curvature►posterior wall, then repeated in the opposite direction. Each U-shaped knot that had six needle run-throughs was defined as one plication. Four plications were performed along the greater curvature, but leaving a space without plication at the gastric fundus, about 3 cm below the esophagogastric juction, in order to create a gastric tubulization similar to that seen in surgical gastric plication (Figure 4). The procedure was finished in 50 min without bleeding or other complications.

After the procedure, the patient was kept in hospital, fasting, with antiemetics and proton pump inhibitors. 

## RESULTS

The 56 year-old male patient with a BMI of 35.17 kg/m^2^ had been treated for the previous two years by the institution's multidisciplinary team and failed clinical/drug treatment for obesity. The patient did not have comorbidities; he was a former smoker and complained of joint pain in the lower limbs. Laboratory and imaging exams were normal, except for an ultrasound image suggesting hepatic steatosis. Previous upper digestive endoscopy was found to be normal and the urease test was negative. 

The patient woke up with mild abdominal pain, without nausea or vomiting. A CT scan and contrast X ray (iodinated contrast) were performed on the day of the procedure, identifying a small pneumoperitoneum and gastric tubulization, but no contrast leakage. The following day a new imaging evaluation was performed with the absence of pneumoperitoneum. Since the patient had no complaints, he was put on a liquid diet without residues and was discharged from the hospital on the second day after surgery. 

## DISCUSSION

Intraluminal endoscopic suturing was initially developed with the goal of treating GERD^4^ ^,^ ^15^. Overstich^(r)^ performs full-thickness suturing, allowing for a serosa to serosa approximation with lasting plication durability. This was evidenced by the presence of pneumoperitoneum after the procedure, which is indicative of gas leakage into the cavity after suturing^7^. In the first-in-human case report, the procedure initially lasted 3.48 h, decreasing to 2 h at the end of the series, after technique improvements^6^. In 2013 Abu Dayyeh et al. demonstrated technical viability and safety in a series of four cases^2^. Other studies have been published in the literature reporting significant weight loss, even in the medium term^1^ ^,^ ^7^ ^-^ ^9^ ^,^ ^14^. 

In a prospective series of 55 patients, six to eight plications were performed in each case, without major complications. During radiological evaluation six months after the procedure, it was found that the stomach's tubular aspect had been preserved. There was a loss of 18.9 kg and 55.3% excess weight^7^.

Lopez-Nava and Galvão Neto et al., in a one-year follow up study including 25 patients, did not observe major complications, with average procedure time of 80 min (50-120). There was a total weight loss of 18.7% after one year, related to a multidisciplinary follow-up, with statistics demonstrating that the number of visits to a nutritionist and psychologist influenced the results. It was possible to perform an endoscopic evaluation one year after the procedure in half (50%) of the patients and radiological evaluation in 80% of patients, showing that the tubular aspect remained after this period. In only one case was it necessary to perform a new procedure due loss of plication^8^. ESG is durable after one year, with possible repetition of the procedure, if necessary, thus reaching weight loss increments. 

In a multicenter evaluation of 242 patients, there was a TBWL of 19.8% after 18 months, with a serious complication rate of 2% - perigastric fluid collection treated with percutaneous drainage and antibiotics, self-limited hemorrhage from splenic laceration, pulmonary embolism, pneumoperitoneum/pneumothorax - without deaths or severe outcomes^10^.

OverStitch^(r)^ has recently been approved by the agency of the Health Ministry, ANVISA, for use in Brazil. The case described in this report is a pioneer in this country. 

The ESG was performed in a total time of 50 min, within the same standard as described in the literature, without bleeding or other complications during the procedure. The patient did not present early complications and continues to be an outpatient attended by a multidisciplinary team. 

ESG is a less invasive and cheaper alternative than conventional bariatric surgery, with promising results, especially when performed with multidisciplinary follow-up. 

## CONCLUSION

The endoscopic sleeve gastroplasty is safe, with acceptable technical viability and reproducibility, with a short procedure time and without early complications.
